# Changes in Energy Expenditure with Weight Gain and Weight Loss in Humans

**DOI:** 10.1007/s13679-016-0237-4

**Published:** 2016-10-13

**Authors:** Manfred J. Müller, Janna Enderle, Anja Bosy-Westphal

**Affiliations:** 1 Institute of Human Nutrition and Food Science, Faculty of Agricultural and Nutritional Sciences, University of Kiel, Düsternbrooker Weg 17, D-24105 Kiel, Germany; 2 Institute of Nutritional Medicine, University of Hohenheim, Stuttgart, Germany

**Keywords:** Energy expenditure, Resting energy expenditure (REE), Total energy expenditure (TEE), Diet-induced thermogenesis (DIT), Activity-related energy expenditure (AEE), Exercise activity thermogenesis (EAT), Non-exercise activity thermogenesis (NEAT), Energy balance, Metabolic efficiency, Body composition, Fat mass, Fat-free mass (FFM), Total body water (TBW), Obesity, Leptin, Insulin, Thyroid hormones, SNS activity, Adaptive thermogenesis (AT), Caloric restriction, Overfeeding, Weight loss, Weight maintenance

## Abstract

Metabolic adaptation to weight changes relates to body weight control, obesity and malnutrition. Adaptive thermogenesis (AT) refers to changes in resting and non-resting energy expenditure (REE and nREE) which are independent from changes in fat-free mass (FFM) and FFM composition. AT differs in response to changes in energy balance. With negative energy balance, AT is directed towards energy sparing. It relates to a reset of biological defence of body weight and mainly refers to REE. After weight loss, AT of nREE adds to weight maintenance. During overfeeding, energy dissipation is explained by AT of the nREE component only. As to body weight regulation during weight loss, AT relates to two different *set points* with a *settling* between them. During early weight loss, the first *set* is related to depleted glycogen stores associated with the fall in insulin secretion where AT adds to meet brain’s energy needs. During maintenance of reduced weight, the second *set* is related to low leptin levels keeping energy expenditure low to prevent triglyceride stores getting too low which is a risk for some basic biological functions (e.g., reproduction). Innovative topics of AT in humans are on its definition and assessment, its dynamics related to weight loss and its constitutional and neuro-endocrine determinants.

## Introduction

Weight loss and weight gain are associated with declines and increases in energy expenditure (EE), which mainly follow changes in the metabolically active component of the body, i.e. fat-free mass (FFM). Most of these changes are non-adaptive and occur passively. However, weight change does not exactly follow prediction based on calculation of energy imbalance. This is explained by FFM-independent metabolic adaptations, i.e. adaptive thermogenesis (AT). AT limits changes in energy stores in response to varying energy intake and/or EE, e.g. AT explained about 50 % of the less-than-expected weight loss in obese patients [1, 2]. Energy sparing (with weight loss) and energy dissipation (with weight gain) are related to the issues of obesity as well as voluntary (e.g. due to dieting in obese subjects) and unvoluntary weight loss (e.g. during cachexia in cancer patients).

AT refers to (i) the resting (or non-activity-related) component of EE including resting energy expenditure (REE), as well as the diet-induced thermogenesis (DIT), and (ii) non-resting component (i.e. activity-related energy expenditure, AEE, which is further divided into exercise and non-exercise activity thermogenesis, EAT and NEAT) of total energy expenditure (TEE). Metabolically, AT has been explained by the ratio of glycolytic to oxidative enzymes together with an altered efficiency of free fatty acid oxidation in skeletal muscle, “futile” cycles consuming ATP without a net change in products (e.g. hydrolysis of triglycerides and subsequent re-esterification in adipocytes), changes in the ATP-costs per muscle contraction, mitochondrial uncoupling in brown adipose tissue, energy-consuming pathways like lipogenesis, NEAT and/or partitioning of energy to fat mass or FFM [3]. These mechanisms are considered to be under genetic and hormonal control, i.e. by insulin, leptin, thyroid hormones and sympathetic nervous system (SNS) activity.

AT has been related to (i) negative energy balance due to caloric restriction and/or excessive exercise, (ii) chronic overfeeding, (iii) re-feeding after weight loss and (iv) weight maintenance after weight reduction. This will be discussed in detail.

(i) With *caloric restriction*, negative energy balance and weight loss cause decreases in all the energy expenditure components, i.e. REE, DIT and AEE (for reviews see [3, 4]). Sixty-five years ago, the Minnesota Starvation Experiment was the first quantitative description of AT in humans [5••]. Since then, AT has been reproduced in experimental and clinical studies on weight loss. AT varied between 100 and 500 kcal/day, it is observed in lean as well as overweight subjects. AT is independent of the weight loss strategy.

(ii) In the 1968 Vermont *overfeeding* study, body weight gain was lower than expected from the excess of energy intake [6]. This had been explained by an increase in EE with overfeeding as a dissipative mechanism to oppose weight gain. The concept was in line with the “gluttony” concept proposed by Miller et al. about 50 years ago [7, 8]. However, throughout the following decades, numerous well-controlled studies could not confirm mass-independent increases in REE and/or DIT during chronic overfeeding (for reviews see [9–15]). With continuous overfeeding, body energy stored was 60–75 % of excess energy leaving the rest for an increase in EE which was explained by obligatory costs (e.g. for gaining body protein, increased cost of walking, etc. [9–11]. These calculations left less than 10 % of overfeeding-induced energy expenditure unexplained which was accounted for errors of methods and assumptions to estimate energy balance questioning energy dissipation. However, during controlled overfeeding, the non-resting component of EE, i.e. EAT increases at unchanged NEAT [9, 16, 17]. This effect was independent of weight gain-associated changes in body composition. Using a highly controlled protocol in lean subjects, intermittent periods of 3 weeks overfeeding at +20, +40 and +60 % of energy needs at controlled and low physical activity increased TEE, while again, there was no evidence for mechanisms able to dispose excess energy [18]. Macronutrient composition may add to metabolic changes in response to overfeeding. While there were no differences in increases in EE after either carbohydrate or fat overfeeding [19], a high protein intake may have a greater effect. Hypercaloric protein-rich diets (with a protein content at 25 % of energy intake) increased FFM, TEE and REE [20•]. However, AT was not different between diets differing in protein content. This is also against the idea that overfeeding low protein diets stimulates thermogenesis [21]. Taken together, during overfeeding, an adaptative increase in REE is non-existent and probably more a measurement artefact [15, 18, 20•, 22]. By contrast, AT seems to be related to the non-resting component of EE [9, 16, 17].

(iii) When recovering from starvation, people spontaneously overeat; body weight and fat mass increase. Weight regain may be forced by persistence of the starvation-induced suppression of thermogenesis [5••, 15, 22]. However, during a controlled caloric restriction-*refeeding* cycle in healthy lean men, mass-independent decreases in REE reversed within 2 weeks of refeeding [5••, 23••]. No energy dissipation occurred with refeeding and weight regain.

(iv) If weight loss-induced AT persists during refeeding, it may carry a long-term risk of “overshooting” pre-starvation body weight and weight loss-weight regain cycles (for a review see [17, 24–26, 27••, 28]). A low REE is a risk of weight gain, and it impedes *weight maintenance* [26, 29]. The role of AT in the control of weight loss maintenance has been addressed in a seminal series of controlled experiments performed by Leibel and Rosenbaum (for reviews see [17, 24, 25•]). These authors investigated normal and overweight subjects after a 10 or 20 % weight loss with a subsequently controlled weight stabilisation phase of at least 14 days [17]. In this protocol, AT was about 50 to 140 kcal/day and mainly related to the non-REE component of TEE.

In a long-term observation study on weight-reduced overweight patients, weight regainers had a reduced AT when compared with weight stable patients [26]. By contrast, after massive weight loss of nearly 60 kg in severely obese patients, weight regain was not associated with AT [27••]. The persistence of AT during weight gain after weight loss has been questioned by controlled feeding experiments [5••, 23••] and computational modelling of human energy metabolism [30]. Following a starvation-refeeding cycle, the curve traced by REE on FFM followed a loop with a decrease in the REE-FFM association induced by negative energy balance and a nearly parallel increase during refeeding: At a similar FFM, REE was lower after 12 weeks of starvation when compared with 12 weeks of refeeding [30].

Weight change-associated changes in EE vary among individuals but may be related to one another within individuals responding to weight gain and weight loss [31, 32]. With negative energy balance, a high AT reduces the drive towards weight loss. By contrast, a low AT in response to overfeeding increases the metabolic drive to gain weight. In a given individual, both mechanisms add to efficient energy use to conserve body energy providing a so-called *thrifty* phenotype [32, 33]. *Vice versa*, a low AT in response to negative energy balance together with a high AT during positive energy balance favours weight loss during caloric restriction and limits weight gain with overfeeding altogether characterising a so-called *spendthrift* phenotype [32, 33]. Over long-term, metabolic phenotypes characterised by AT are correlated to subsequent weight changes.

Based on the quantitative physiology of weight change, robust mathematical (so-called “flux-balance”) models of human energy metabolism and AT have been developed and validated [30, 34•, 35]. Crucial points of these models relate to (i) energy partitioning (i.e. energy imbalance is divided between fat mass and FFM) and (ii) possible feedbacks of fat mass and/or FFM on energy intake which are assumed to be constant. Computational modelling is now widely used to predict weight changes in obese patients during dieting or to explain the non-linear function of weight loss.

Taken together, the present data suggest an asymmetry in AT of the resting component of EE in response to changes in energy balance. While there is no adaptation in the REE component of EE in response to over-feeding and refeeding, REE-related AT refers to weight loss only. In addition, adaptation in the non-resting component of EE relates to maintenance of reduced body weight as well as possible energy dissipation with overfeeding. During negative energy balance AT results from a compensatory feedback directed towards conservation of energy and limitation of weight loss. AT has been related to biology of body weight regulation and is a possible “re-*set*” of defence. From a clinical standpoint, AT is seen as a metabolic vulnerability of obese patients.

## New and Interesting Findings

AT seems to be well-established in the biology of weight loss. Presently, there are three innovative topics related to AT in humans: first, definition and assessment of AT; second, the dynamics of metabolic adaptation related to weight loss and weight loss maintenance; and third, the determinants of AT.

## Definition and Assessment of AT

Following Ancel Keyes original definition [5••], AT refers to changes in REE which are independent from FFM *and* FFM composition. During weight loss, AT reflects decreases in specific metabolic rates of organs and tissues within FFM. It follows that AT can only be assessed based on accurate body composition analysis (BCA) including magnetic resonance imaging (MRI) to quantitate masses of low (i.e. skeletal muscle) and high metabolic rate organs (i.e. brain, liver, heart and kidneys) [3, 36, 37••]. After adjustment of REE for FFM, only AT was calculated to be around 100 kcal/day (or about 50 % of the fall in REE; 23). In that study, FFM decreased by 2.4 kg with concomitant changes in the masses of skeletal muscle (−1.5 kg) and liver (−0.2 kg) [23••]. Adjusting REE for changes in FFM plus the anatomical composition of FFM, “true” AT was calculated to be 70 kcal/day [23••]. It is tempting to speculate that additional adjustments of REE for the molecular composition of organs and tissues (e.g. for their hydration, protein content and density) will further affect the calculation of AT [36].

Normalising REE for FFM or FFM + fat mass has limitations when one addresses changes in REE with weight loss. Normalisation is a statistical approach. It does not take into account physiological changes in adipose tissue-derived FFM (water, protein, minerals in adipocytes), the proportion of the high metabolically active organs and tissues to FFM (see above) and a yet poorly defined thermic effect of adipocytes probably due to their secretory and inflammatory activities [3, 37••]. The characteristics of regressions for FFM and fat mass differ between different degrees of adiposity [37••, 38]. In fact, applying the REE on FFM + FM-relationship before weight loss to data obtained after a 6-kg weight loss led to an error of about 70 kcal/day. The overestimation of AT calculated from the REE vs FFM + FM relationship before weight loss increases after a 50-kg weight loss in severely obese subjects explaining (but also questioning) a magnitude of AT of about 300 kcal/day at a decrease in REE of about 600 kcal/day [39]. By contrast, using a %fat mass-specific regression equation to normalise REE before and after weight loss reduced AT to 120 kcal/day [37••].

AT can be calculated from the difference between REE adjusted for FFM + fat mass before and after weight loss. Alternatively, the difference between measured REE and REE calculated from organ and tissue masses times their specific metabolic rates can be used [23••, 37••, 40, 41]. This difference is a measure of the mass-independent changes in energy expenditure, and it increases after weight loss. However, the approach depends on some assumptions, e.g. regarding the molecular composition of organs and tissues and their specific metabolic rates. Since 3 days of caloric restriction may result in losses of up to 2–3-l water with an accompanying 3.6 % change in FFM hydration [36], a weight loss-associated change in FFM does not necessarily reflect a decrease in metabolically active FFM. Overestimating the loss of metabolically active FFM results in an underestimation of AT [22, 36]. In addition, specific metabolic rates of individual organs and tissues are not constant but vary with adolescence, age above 55 years, obesity, weight change and work load [41, 42].

There is need of more sophisticated concepts to assess AT. Presently, detailed BCA at the organ-tissue level (using whole body MRI to assess organ and tissue masses) together with BCA at the molecular level (using balance and dilution techniques to assess changes in tissue hydration and protein content) is needed for proper adjustments of REE. In addition, direct estimates of specific metabolic rates of organs and tissues by magnetic resonance spectroscopy and/or positron emission tomography (PET) will add to assess functional body composition and AT. Faced with these conceptual and methodological caveats, AT cannot be considered as a biological entity. In addition, scientists should be honest about the limits of detection using state-of-the-art technologies to assess EE [22, 43]. The precision differs between assessments of REE by indirect calorimetry (between 1 and 3 %) and measurements of TEE with doubly labelled water (DLW, above 5 %) where the intra-individual variance may exceed the inter-individual variance [22].

## Dynamics of Metabolic Adaptation Related to Weight Loss and Weight Loss Maintenance

Changes in energy expenditure are functions of time with fluctuations within minutes, hours, days, weeks and months. In the Minnesota Starvation Experiment, REE and body composition were measured after 1, 3 and 6 months of caloric restriction [5••]. This time scale is in line with more recent clinical studies on AT which vary in observation periods between 3 weeks and 6 years ([1, 27••, 44–49]; for reviews see [3, 4, 50]). Weight loss results from a negative energy balance and changes in body composition; it is not continuous but curve-linear ending when a new steady state and, thus, a new equilibrium between energy intake and energy expenditure are reached [51].

During caloric restriction, the first phase of weight loss is rapid and lasts less than a week (phase 1); this is followed by a second phase characterised by a slower weight loss (=late phase) [51]. With total starvation, phase 2 continues until fat mass is nearly completely depleted. Weight loss becomes deleterious to the subject when body protein is the only endogenous energy source left. During controlled 3-week semi-starvation, healthy young men lost about 3.2 kg during week 1 and 1.3 to 1.4 kg/week during week 2 and 3 [23••, 36, 40]. Decay constants in weight were calculated as *K*_1_ = −0.78 kg/day (week 1 = phase 1) and *K*_2_ = −0.19 kg/day (week 2 and 3 = phase 2). As to body composition, the corresponding decreases in FFM were 313, 90 and 66 g/day, respectively [23••]. Concomitantly, fat mass decreased by 168, 109 and 142 g/day.

Weight loss is characterised by defined changes in body composition. These changes relate to AT. During phase 1, decreases in FFM exceed the decrease in fat mass; they are due to losses in intracellular (due to glycogen and protein mobilisation) and extracellular water (due to an increase in natriuresis) rather than to losses in cell mass per se [23••, 36]. By contrast, phase 2 relates more to the steady and ongoing loss of fat mass. Since the energy content per kilogram change differs between body fat (i.e. 9434 kcal) and FFM (i.e. 1815 kcal), the composition of weight lost is the main determinant of weight changes in response to a given energy balance [34•, 35, 51]. While decreases in fat mass explained 34 % of weight loss during phase 1, this proportion increased to 64 and 68 % during phase 2. Thus, the energy content per kilogram weight change increased from 4409 kcal during phase 1 to 5209 and 7105 kcal during phase 2 [23••].

Obviously, assessment of AT must follow the dynamics of weight loss. EE should be measured longitudinally before and during the two different phases of weight loss. Unfortunately, this idea has not been addressed in nearly all of the above-mentioned clinical studies on AT where EE had been measured before and after weeks or even after months or after years of weight loss only. During controlled caloric restriction, AT becomes manifest within the first 3 days with no further changes during phase 2 [23••, 40]. This is evidence for the idea that regulation of AT occurs during phase 1. By contrast, the decrease in REE closely followed weight loss during phase 2 (i.e. there was no further mass-independent adaptation in REE). Faced with these dynamics clinical investigations cited above did not address the regulation of AT. All the worse, many clinical studies on weight loss are metabolically uncontrolled, i.e. it remains unclear whether weight-reduced patients have reached a weight stable state or whether they are still losing or already regaining body weight.

## Determinants of AT

**Fig. 1:**
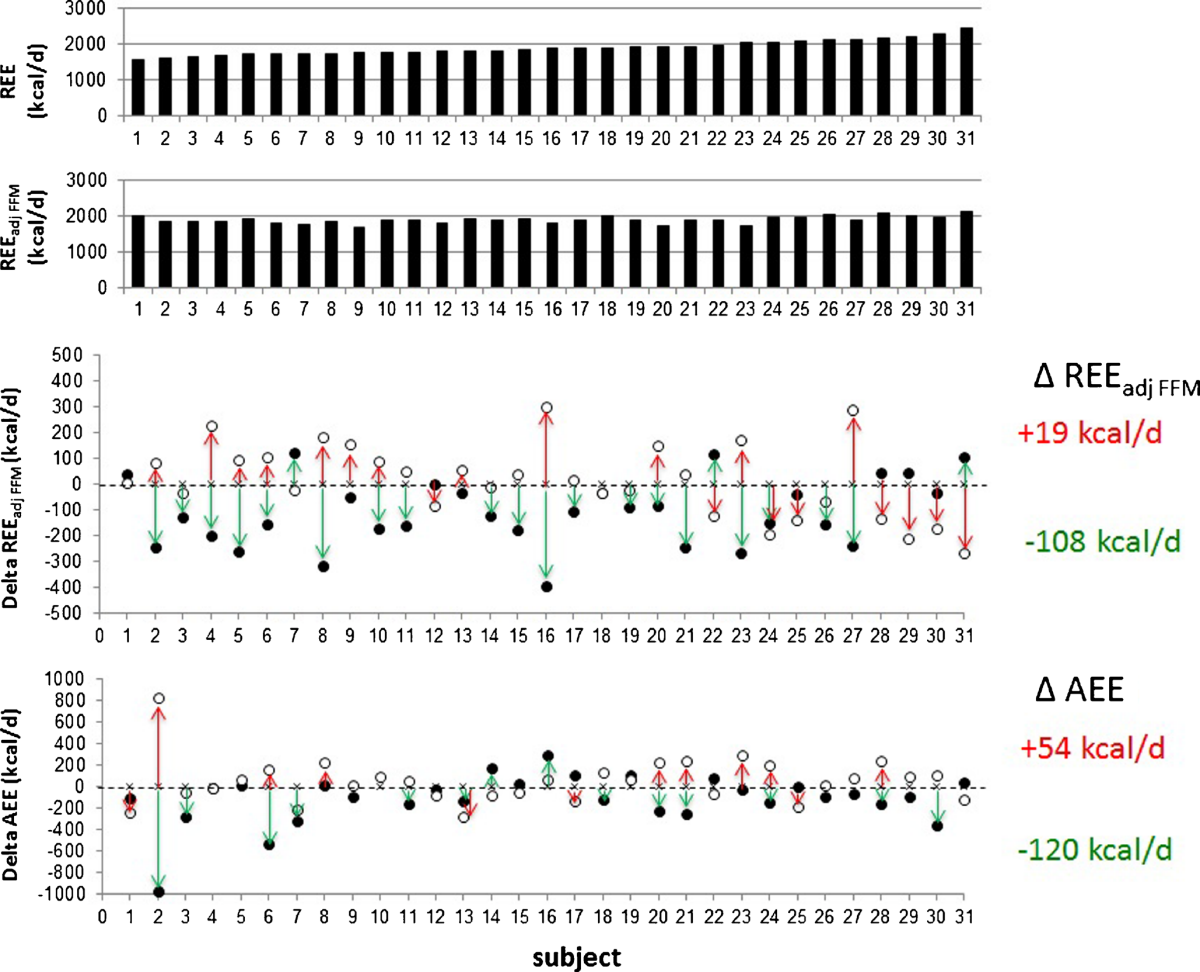
Inter-individual variances in the resting (=ΔREE_adj FFM_) and non-resting compartment (ΔAEE_adj FFM_) of adaptive thermogenesis (AT) during controlled 3 weeks under-feeding (at −50 % of energy requirements in *green*) and 2 weeks re-feeding (at +50 % of energy needs in *red*) protocol in 31 healthy and normal weight young men. For original data see ref 23. The subjects were ranked according their REE before intervention (*upper panel*). After adjustment for FFM, there were no interindividual differences in REE measured before the weight cycle. Mean group changes with weight loss and weight gain are given on the *right side of the figure*. AT occurred at underfeeding only. *REE* resting energy expenditure, *AEE* activity-related energy expenditure, *FFM* fat free mass (colour figure online)

AT is considered as an outcome of autoregulatory control that operates to limit weight loss and to restore body composition [3, 14, 22, 52, 53]. During weight loss, there is a considerable inter-individual variance in AT [3, 23••, 44, 54•] (Fig. 1). Taking into account the precision of indirect calorimetry, up to 60 % of subjects experienced a greater-than-expected decline in REE after weight loss [3, 23••]. On the other hand, AT was found to be reproducible [23••]. Thus, AT is considered as an individualised trait.

**Fig. 2:**
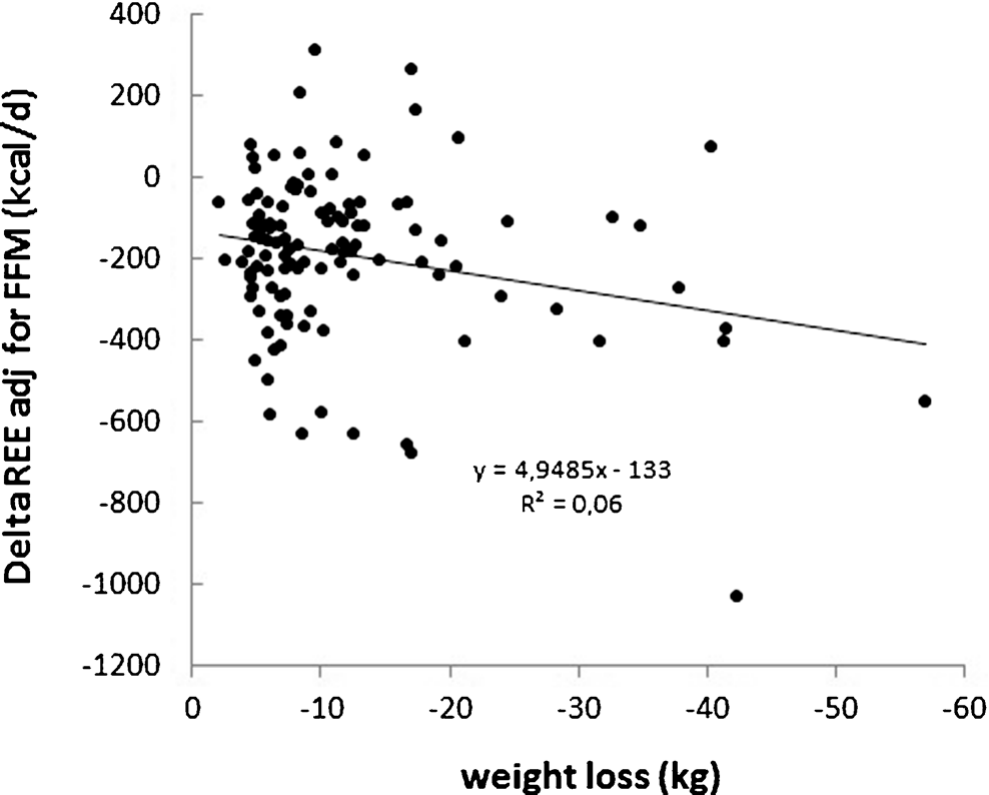
Association between adaptive thermogenesis and weight loss in 151 overweight patients after dietary or bariatric surgery-induced weight loss. Data from refs 1, 55, and 56

AT was independent of macronutrient composition of the diet [44–47]. It was thought to be proportional to the degree of weight loss with an additional effect of baseline weight [47]. However, analysing data on obese patients before and after weight loss [1, 55, 56], the association between weight change and AT was weak (see Fig. 2). This was in line with the results of a controlled clinical study on obese patients using total starvation or different hypocaloric diets for weight loss [48]. By contrast, the starvation-induced fall in REE was closely related to REE before weight loss: The higher baseline REE was, the higher was AT [23••].

In addition, there is an association between AT of the non-REE component of EE and maintenance of reduced body weight [24, 25•, 52]. Most of AT occurred during weight maintenance after a moderate weight loss of −10 % with smaller additional effects induced by a weight loss of −20 % [17, 52]. Recently, Rosenbaum and Leibel proposed three models of AT during weight maintenance [25•]: Model 1, no AT, i.e. a “mechanical model” related to settling of body weight; model 2, fixed AT due to a threshold response (model 2) and model 3, AT is proportional to weight loss. With initial weight loss, both REE and AEE decreased by a fixed amount of kilocalories. This was independent of changes in body composition. With further weight loss, only AEE continued to decrease. Thus, AT related to the resting compartment of TEE followed the threshold model, whereas AT in the non-resting compartment of TEE is proportional to weight loss [25•].

Determinants of AT have to be discussed in the contexts of (i) weight loss and (ii) maintenance of reduced weight.

### Weight Loss

Regulation of AT has been related to changes in the composition of FFM (i.e. a change in the proportion of high metabolic rate organs to muscle mass as well as tissue hydration), reduced endocrine signals from triiodothyronine (T3), insulin and SNS activity and/or a reduced feedback from adipocytes brought about by a fall in leptin secretion.

In a clinical study, obese women lost a mean of 9.5 kg body weight with an AT of 112 kcal [55]. Concomitantly, the relative loss of high metabolic rate organ masses was significantly higher than was the change in low metabolically active parts of FFM [55]. Changes in FFM composition explained about 50 % of AT. Comparing patients with high vs low AT, the former better conserved liver and kidney masses with weight loss [55]. During controlled underfeeding, AT was 108 kcal; 36 kcal was explained by changes in FFM composition [23••]. There were no associations between AT and changes in fat mass or regional fat depots [23••, 55].

Although fat mass, plasma levels of leptin, leptin per fat mass and T3 as well as SNS activity all decreased with negative energy balance and weight loss, neither their levels nor their changes were associated with AT [23••]. In severely obese subjects who had lost about 40 % of their body weight, both the decreases in plasma leptin levels and REE exceeded the loss in body weight, but no associations were detected between changes in EE, and changes in leptin concentration [39]. Although some authors have found some correlations between the weight loss-associated decreases in plasma leptin levels and AT, leptin explained 6 % of the variance in AT only [49, 57]. Investigating AT up to 12 months after bariatric surgery with a group mean weight loss of 44 kg showed that metabolic adaptation correlated with changes in insulin, adiponectin, leptin, T3, gut hormones and SNS activity [47]. By contrast, there was no correlation between AT and changes in plasma leptin levels in the participants of the “Biggest Looser Competition” with mean AT of 275 kcal/day after 7.5 months and 499 kcal/day after 6 years and concomitant loss and regain of fat mass of −47.2 and +35.2 kg, respectively [27••]. Concomitantly, plasma leptin levels decreased and increased by −38.6 and +25.12 ng/ml.

In addition, there were no associations between AT and the weight loss-associated decrease in circulatory T3 [23••, 27••] and SNS activity [23••]. This is not against the findings that low-dose T3 or catecholamine replacements increase REE [58, 59]. However, a drug replacement protocol may not resemble physiological regulation. In fact, pharmacological inhibition of endogenous production of T3 in isocalorically fed subjects was without effect on energy expenditure [60]. Taken together, leptin, T3 and SNS-activity seem to be unrelated to AT.

Since regulation of AT occurred during phase 1 of weight loss, one has to address its possible determinants within this period. During phase 1, AT was associated with the starvation-induced fall in insulin secretion, low RQ, negative fluid balance with losses in intracellular to extracellular water and a low free water clearance rate (FWCR; [23••]). Depletion of hepatic glycogen stores, losses in ICW and ECW (due to increased natriuresis) all relate to the starvation-induced fall in plasma insulin concentrations [61, 62]. Whole body RQ decreased by 8 or 11 % in response to semistarvation or total fast, respectively [23••, 62]. Within 3 days of total fast, the rate of hepatic glycogenolysis decreased by 93 % with a related decrease in total liver glycogen content from 373 to 47 mmol [62]. This resulted a decrease in liver volume by 370 ml with fasting [62] compared with 150 ml during semistarvation [23••]. Concomitantly, whole body fluid balance became negative by 570 ml/week [23••]. The lower insulin secretion was, the lower FWCR was. A low insulin secretion and a reduced FWCR were both associated with a high AT and *vice versa*. By contrast, there were no associations between AT and changes in urinary 24-h sodium and aldosterone excretion as well as plasma levels of natriuretic peptides [23••].

AT was associated with starvation-induced decreases in body temperature, heart rate and glomerular filtration rate [23••] suggesting that AT is part of the concerted physiological response to weight loss with the fall in insulin secretion as its major characteristic. This is in line with clinical data that high AT correlated with the decrease in plasma insulin levels [55].

During phase 1, AT is explained by “threshold model” related to the fall in insulin secretion [25•, 40]. During phase 2, there is no additional effect of ongoing weight loss on the REE-FFM regression line. This is evidence for a “mechanical model” [25•].

### Weight Loss Maintenance

During maintenance of reduced body weight, TEE remained reduced [24]. This relates to AT in the non-resting component of EE; it is accompanied by increased skeletal muscle work efficiency, decreased plasma levels of leptin and T3 associated with low SNS activity [23••, 63]. Decreases in fat mass were related to changes in TEE [24, 25•, 52, 57]. This is in line with the concept that after weight loss AT is a direct function of body fat depletion [28]. The loss in fat mass then generates the fall in plasma leptin concentrations which is related to AT. In fact, replacing leptin during maintenance of reduced weight reversed two-thirds of the fall in TEE and AEE while increasing work efficiency in skeletal muscle [64, 65•]. When compared to a placebo-treated group, TEE adjusted for FFM (but not REE) was significantly greater in “leptin-treated 10 % weight-reduced weight-maintained patients” suggesting that higher leptin levels promote weight loss [65•]. Leptin replacement reversed the neuroendocrine adaptations and increased in plasma T3 levels and SNS activity suggesting that the non-resting component-related part of AT is under endocrine control. Adaptation of AEE follows the dynamics of weight loss and thus supports model 3 of energy homeostasis [25•]. It should be mentioned that controlling body weight after gradual weight loss (as done in ref) [17, 64, 65•] does not follow a physiological response but may interfere with biology. This situation may be considered as an exogenously “forced” regulation.

One of the caveats of the adipocentric view of AT is that leptin itself had no effect on EE in leptin deficient or overweight patients. Giving recombinant leptin to children with congenital leptin deficiency (an extreme situation producing a severely obese phenotype), for weight loss in obese patients or for weight maintenance in obese patients after gastric bypass surgery was without effect on TEE and REE [66–69]. By contrast, leptin replacement in patients with generalised lipodystrophy (characterised by the absence of subcutaneous fat, fatty liver and marked hypoleptinemia which is again an extreme phenotype) decreased rather than increased REE with relatively minor decreases in body weight [70].

In population studies on subjects differing in body weight, normal or elevated plasma leptin levels did not correlate with energy expenditure [71, 72]. By contrast, there was a close association between very low leptin levels and REE in severely underweight patients with anorexia nervosa; this association disappeared again during treatment, i.e. with increases in fat mass and plasma leptin concentrations [71]. These observations fit to the idea of an asymmetric leptin physiology [63, 73]: Leptin has no thermogenic effect at normal or high concentrations, whereas the effect becomes apparent after leptin levels fall below a threshold level invoking a mass-independent decrease in EE. This threshold may reflect a minimum level of fat mass needed for biological functioning.

However, when compared with underweight anorectic women, weight loosing obese patients or even normal weight men are unlikely to reach the minimum threshold of fat mass and/or leptin levels. Accordingly, an alternative threshold shift paradigm has been proposed for obese patients [63, 74]. Comparing the association between BMI and plasma leptin levels in 110 patients undergoing bariatric surgery, the slope of the relation was flatter after surgery. The authors took this as evidence for a weight threshold which may trigger the leptin response to defend body weight. Energy expenditure was not measured in that study, but the idea of a shift to an elevated threshold has also been proposed for obese compared to normal weight subjects to explain their defences of higher fat stores [74, 75•].

Taken together, there was no thermogenic effect of leptin in subjects at usual weight and during weight loss. By contrast, an effect of exogenous leptin administration was seen in weight-stable-weight-reduced subjects only [64, 75•]. To reach weight maintenance, leptin was substituted to the pre-weight loss plasma level. Thus, with replacement circulating leptin probably increased from values below to above the individual thresholds waning the effect of leptin on energy expenditure and energy balance. Leptin thresholds may differ between different metabolic functions (e.g. for energy intake vs energy expenditure) [75•] and may also relate to thresholds of other hormone-function relationships being part of the concerted neuroendocrine responses to negative energy balance, weight loss and weight maintenance. These thresholds are considered as individualised traits adding to the variance in AT as well as long-term weight changes [25•].

## Conclusions: A Proposed Concept and a Future Perspective

**Fig. 3:**
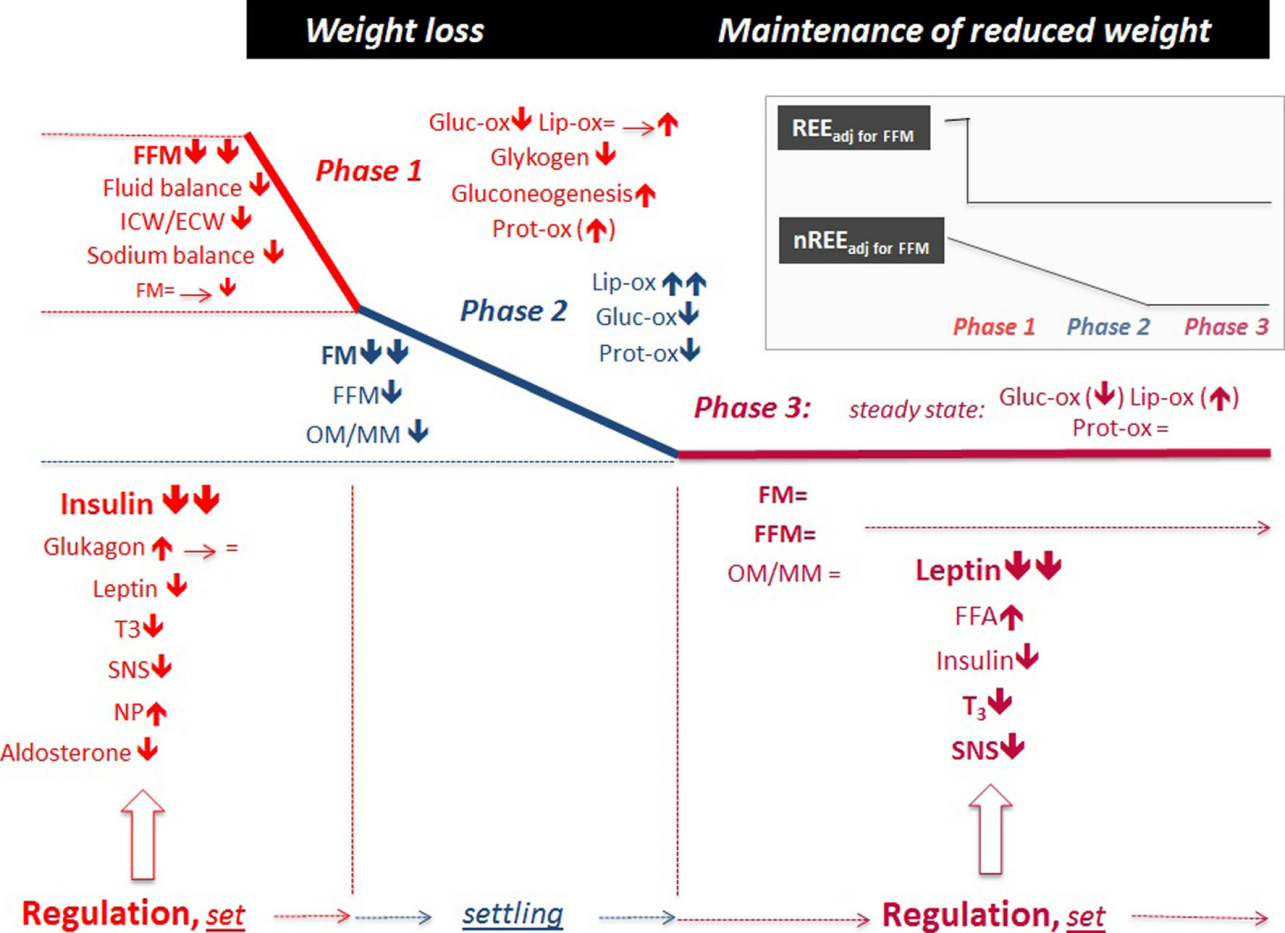
Overview about metabolic adaptation with weight loss and during maintenance of reduced body weight. During the first week of caloric restriction, adaptive thermogenesis (AT) relates to the resting component of energy expenditure (REE) and follows the depletion of hepatic glycogen stores due to the immediate fall in insulin secretion. Mobilisation of glycogen is associated with changes in fluid balance and fat free mass. AT is seen as an immediate adaptation to negative energy balance as part of body weight regulation according to a *set point*. With ongoing underfeeding and weight loss, phase 2 is characterised by a loss of fat mass which follows the negative energy balance up to a *settling point* where a new steady state is reached. Then, maintenance of reduced body weight (phase 3) is due the degree of reduced fat mass and low leptin levels associated with a low T3 state and low SNS activity. This endocrine pattern carries the risk of weight regain. The inserted graph on the right shows that during phase 1, AT is characterised by an adaptation of the resting component of energy expenditure. This is maintained throughout further weight loss and during successful maintenance of reduced body weight. By contrast, adaptation in the non-resting component of energy expenditure (nREE) is proportional to weight loss. Up to now, early changes in nREE have not been investigated, and data are available after 3 weeks of semistarvation only [23]. *FFM* fat free mass; *ICW* intracellular water; *ECW* extracellular water; *FM* fat mass; *OM* masses of high metabolically active organs = sum of masses of brain, heart, liver, and kidneys; *MM* skeletal muscle mass; *FFA* free fatty acids; *T3* tri-iodothyronine; *SNS* sympathetic nervous system activity; *NP* natriuretic peptides; *Gluc-ox* glucose oxidation rate; *lip-ox* lipid oxidation rate; *prot-ox* protein oxidation rate. See text for further details and references

With weight loss, AT has to be differentiated in relation to (i) the individual component of TEE, (ii) different phases of weight loss and (iii) weight loss maintenance. The first issue is about resting and non-resting EE. The second is about hepatic glycogen mobilisation and mass-independent sparing energy with negative energy balance. The third issue relates to a possible “reset” of the biological defence of body weight after weight loss and thus the risk of weight regain. Figure 3 surveys metabolic adaptation to weight loss and weight maintenance and their determinants with time. We propose to differentiate between three phases of weight loss addressing two different regulatory systems. The first is the immediate control of metabolism in response to negative energy balance (phase 1), whereas the second regulatory system impacts weight maintenance after weight reduction (phase 3). Between the two regulations (i.e. during phase 2), changes in EE are proportional to weight change, and there is no further increase in AT with ongoing weight loss.

During negative energy balance, mitochondrial carbon load decreases and ATP demand is met by the mobilisation of endogenous sources, i.e. glycogen and triglycerides. Losses in functional body mass and AT add to reduce ATP demand. Metabolically, the paramount characteristic of phase 1 is the depletion of glycogen stores, whereas in phase 3, AT is triggered by the loss in body fat. As far as *set points* (i.e. feedback systems designed to match a target) and *settling points* (i.e. control systems without a *set point*) in body weight regulation are concerned [76, 77], the data give rise to the idea that (i) AT is part of body weight regulation and (ii) there are two *set points* with a *settling* between them. The first *set point* is about the decrease in glycogen stores only. We assume that in early starvation, lower thresholds of (i) liver glycogen or (ii) negative fluid balance associated with glycogen depletion trigger AT. It is tempting to speculate that this early response is related to energy needs of the brain (i.e. brains metabolism requires 80 to 100 g glucose per day). By contrast, AT does not occur after glycogen depletion in response to an isocaloric ketogenic diet and moderate weight loss [78]. This may be explained by concomitant increases in plasma ketones providing an alternative fuel to the brain and, thus, put the glycogen threshold into perspective. During weight maintenance, a lower margin of the leptin-fat mass-association is the second *set point* keeping energy expenditure low to prevent triglyceride stores getting too low which again would limit basic biological functions (e.g. reproduction).

Future biomedical research on the physiology, genetics, cellular and/or molecular aspects of body weight regulation and AT needs robust concepts taking into account conceptual issues, advanced methodological approaches, the dynamics of weight change and *in depth* physical and metabolic phenotyping. We still lack an integrated understanding of AT. It is also worthwhile to remember that our present knowledge about AT in humans is based on a small number of controlled experimental studies (including the nearly 70 years old Minnesota Starvation Experiment; 5) as well as a greater number of more or less uncontrolled clinical observations. Both approaches do not address the “real” physiology of weight changes which may take several years to obtain a new steady body weight [79].
